# Supplementary material for: Primate TRIM5 proteins form hexagonal nets on HIV-1 capsids
Source: eLife. 2016 Jun 2;5:e16269. doi: 10.7554/eLife.16269 (PMC4936896; doi:10.7554/eLife.16269)
Supplement: Supplementary file 1. — (A) List of plasmids used in this study. (B) TRIM5 constructs and their molecular weights. DOI: http://dx.doi.org/10.7554/eLife.16269.020 [file elife-16269-supp1.docx]

**SUPPLEMENTARY FILE 1**

**Supplementary file 1A. List of plasmids used in this study**

| **Plasmid Name** | **Source**^a^ | **Internal ID (WISP#)** | **Addgene ID** | **Backbone** | **Generation** | **Tag** |
| --- | --- | --- | --- | --- | --- | --- |
| **Bacterial Vectors** |  |  |  |  |  |  |
| pET3a-OSF-CypA | [b] | 14-101 | 79039 | pET3a | Quikchange | OSF |
| pET11a-CA_A14C,E45C_ | [c] | 14-106 | 79045 | pET11a | Quikchange |  |
| pET11a-CA_A14C,E45C, A92E_ |  | 14-107 | 79046 | pET11a | Quikchange |  |
|  |  |  |  |  |  |  |
| **Baculovirus Vectors** |  |  |  |  |  |  |
| pDONR221-OSF-TRIM5-21R | [d] | 10-430 | 79063 | pDONR221 | Gateway | OSF |
| pDONR221-OSF-TRIM5-21R_ΔSPRY(1-300)_ |  | 11-546 | 79029 | pDONR221 | Gateway | OSF |
| pFastBac1-OSF-TRIM5α_AGMpyg (1-293)_ | AAW72440 | 13-31 | 79030 | pFastBac1 | SLIC | OSF |
| pFastBac1-OSF-TRIM5α_AGMpyg_ | AAW72440 | 13-53 | 79031 | pFastBac1 | SLIC | OSF |
| pFastBac1-OSF-TRIM5α_CPZ_ | AAV91977 | 13-54 | 79032 | pFastBac1 | SLIC | OSF |
| ­pFastBac1-OSF-TRIM5α_rh_ | DQ842021 | 13-56 | 79033 | pFastBac1 | SLIC | OSF |
| pFastBac1-OSF-TRIM5α_hu_ | NP_149023 | 13-57 | 79034 | pFastBac1 | SLIC | OSF |
| pFastBac1-OSF-TRIMCyp | AAT73777 | 13-63 | 79035 | pFastBac1 | SLIC | OSF |
| pFastBac1-OSF-TRIMCyp_K283D,Q287D_ | AAT73777 | 13-65 | 79036 | pFastBac1 | SLIC | OSF |
| pFastBac1-OSF-TRIM5α_hu, R332P_ | NP_149023 | 15-92 | 79037 | pFastBac1 | SLIC | OSF |
| pFastBac1- Kozak-TRIM5α-FOS | DQ842021 | 15-103 | 79038 | pFastBac1 | SLIC | FOS |
|  |  |  |  |  |  |  |
| **Mammalian Vectors** |  |  |  |  |  |  |
| CSII-IDR2-TRIM5α_AGMpyg_-FOS | AAW72440 | 16-78 | 79064 | CSII-IDR2 | Nhe1-Xho1 | FOS |
| CSII-IDR2-TRIM5α_rh_-FOS | DQ842021 | 16-79 | 79059 | CSII-IDR2 | Nhe1-Xho1 | FOS |
| CSII-IDR2-TRIM5α_CPZ_-FOS | AAV91977 | 16-80 | 79065 | CSII-IDR2 | Nhe1-Xho1 | FOS |
| CSII-IDR2-TRIM5α_hu_-FOS | NP_149023 | 16-81 | 79066 | CSII-IDR2 | Nhe1-Xho1 | FOS |
| CSII-IDR2-TRIMCyp-FOS | AAT73777 | 16-82 | 79067 | CSII-IDR2 | Nhe1-Xho1 | FOS |
| CSII-IDR2-TRIMCyp_K283D, Q287D_-FOS | AAT73777 | 16-83 | 79068 | CSII-IDR2 | Nhe1-Xho1 | FOS |
|  |  |  |  |  |  |  |
| **Virus Production Vectors** |  |  |  |  |  |  |
| phLOX-GFP | [e] | 14-108 |  |  |  |  |
| pCMV-ΔR8.2 | [f] | 04-151 |  |  |  |  |
| pCMV-ΔR8.2-CA_A14C,E45C_ |  | 14-104 | 79047 | pCMV-ΔR8.2 | Quikchange |  |
| pCMV-ΔR8.2-CA_A14C,E45C,A92E_ |  | 14-105 | 79048 | pCMV-ΔR8.2 | Quikchange |  |
| phCMV-VSV-G | [g] | 06-136 |  |  |  |  |

1. Source refers to the GeneBank accession numbers or original papers [b-f] for the description of these constructs or inserts.
2. OSF- sequence was inserted upstream of CypA sequence into the pET3a-CypA (WISP94-01)([Yoo et al., 1997](#_ENREF_8)) designed previously by Quikchange Site-Directed mutagenesis.
3. Plasmid was provided by Dr. Owen Pornillos ([Pornillos et al., 2010](#_ENREF_3); [Pornillos et al., 2009](#_ENREF_4)).
4. ([Ganser-Pornillos et al., 2011](#_ENREF_1)).
5. Plasmid was provided by Dr. Didier Trono ([Salmon et al., 2000](#_ENREF_5)).
6. Plasmid was provided by Dr. Didier Trono ([Naldini et al., 1996](#_ENREF_2)).
7. Plasmid was provided by Dr. [François-Loïc Cosset](http://cvscience.aviesan.fr/cv/883/francois-loic-cosset) ([Sandrin et al., 2002](#_ENREF_6); [Yee et al., 1994](#_ENREF_7)).

**Supplementary file 1B. TRIM5 constructs and their molecular weights**

| **Constructs** | **Expected Molecular Weight (Da)** | **Observed Molecular Weight (Da)** | **Average Yield (mg/L of culture)** |
| --- | --- | --- | --- |
| ΔOSF-TRIM5α_rh_ | 57457 | 57451 | 2.1 |
| TRIM5α_rh_-ΔFOS | 57942 (+AEBSF^a^) | 57940.3 | 1.3 |
| ΔOSF-TRIM5α_AGMpyg_ | 59238 | 59238.1 | 9.6 |
| ΔOSF-TRIM5α_AGMpyg ΔSPRY (1-293)_ | 34504 | 34499.4 | 5.8 |
| ΔOSF-TRIM5α_CPZ_ | 56397(+AEBSF^a^) | 56396.04 | 4.3 |
| ΔOSF-TRIM5α_hu_ | 56492 | 56488.8 | 2.2 |
| ΔOSF-TRIM5α_hu,R332P_ | 56433 | 56430.7 | 3.8 |
| ΔOSF-TRIMCyp | 59649(+AEBSF^a^) | 59654.1; (39113; 38714.1)^b^ |  |
| ΔOSF-TRIMCyp_K283Q,Q287D_ | 54058 | 54056.0 | 5.6 |
| OSF-TRIM5-21R^c^ | 62279 | 62279.4 | 0.5 |
| ΔOSF-TRIM5-21R_ΔSPRY(1-300)_ | 34739 | Not measured^d^ | ~10 |

1. Protein was covalently modified with serine protease inhibitor 4-(2-aminoethyl) benzenesulfonyl fluoride (AEBSF).
2. Proteolytic fragments of TRIMCyp identified by ESI-MASS.
3. ([Ganser-Pornillos et al., 2011](#_ENREF_1))
4. DNA sequence verified by Sanger sequencing
